# Crosstalk between cancer‐associated fibroblasts and immune cells in cancer

**DOI:** 10.1111/jcmm.14745

**Published:** 2019-10-23

**Authors:** Yuanyuan An, Fengtian Liu, Ying Chen, Qing Yang

**Affiliations:** ^1^ Department of Gynecology and Obstetrics Shengjing Hospital of China Medical University Shenyang P.R. China

**Keywords:** cancer, cancer‐associated fibroblasts, immune cells, tumour microenvironment

## Abstract

Multiple studies have shown that cancer‐associated fibroblasts (CAFs) play an important role in tumour progression, including carcinogenesis, invasion, metastasis and the chemoresistance of cancer cells. Immune cells, including macrophages, natural killer cells, dendritic cells and T cells, play a dual role in the tumour microenvironment. Although increasing research has focused on studying interactions between distinct cells in the tumour microenvironment, the complex relationships between CAFs and immune cells remain unclear and need further study. Here, we summarize our current understanding of crosstalk between CAFs and immune cells, which may help clarify their diagnostic and therapeutic value in tumour progression.

## 1 INTRODUCTION

In solid tumours, the major cell types include mesenchymal cells and fibroblasts, which are also known as cancer‐associated fibroblasts (CAFs).^1^, ^2^ In the matrix of normal tissue, fibroblasts aid with tissue repair.^3^ However, CAFs are actually defined as an assembly of heterogeneous mesenchymal cells, whose function may be different from resident tissue fibroblasts.^4^ Cancer‐associated fibroblasts have been isolated from various tumours, such as prostate cancer, lung cancer, breast cancer, gastric cancer, colorectal cancer and pancreatic cancer, whereas CAFs are relatively rare in brain cancer, ovarian cancer and kidney cancer.^5^, ^6^, ^7^, ^8^, ^9^, ^10^, ^11^, ^12^, ^13^ Interactions between cancer cells and the tumour microenvironment (TME) are well known to contribute to the outcome of tumours. Paget's “seeds and soil” theory was proposed more than a century ago. The molecular characteristics of “seeds” (cancer cells) have been fully analysed, but the “soil” (microenvironment) requires further analyses.^14^ Inflammatory cells, vascular cells and fibroblasts are the main components of the “soil”, and these stromal cells usually interact with cancer cells to achieve specific phenotypes. Early studies of fibroblasts focused on wound healing. Once the would heals, the number of activated fibroblasts decreases dramatically.^15^ Compared to the quiescent fibroblasts, activated fibroblasts could express de novo α‐smooth muscle actin (α‐SMA), increase the production of proinflammatory cytokines and cyclooxygenase‐2 (COX‐2), which further mediated the inflammatory response and tumour progression.^16^ In the permanent activation state, fibroblasts promote the growth and progression of tumours, which can affect the behaviour of tumours and patient prognosis.^17^

Increasingly more attention has been given to the TME in recent years. As an important component of the TME, CAFs interact with other cells and secrete various soluble factors through various paracrine mechanisms.^18^ In addition to CAFs, immune cells, eg T lymphocytes, macrophages and natural killer (NK) cells, among others play an important role in the TME. Increasingly more research has focused on studying interactions between CAFs and distinct immune cells in cancer. Takahashi et al found that CAFs, compared with normal fibroblasts (NFs), expressed higher levels of interleukin‐6 (IL‐6), chemokine (C‐X‐C motif) ligand 8 (CXCL8), tumour necrosis factor (TNF) and vascular endothelial growth factor A (VEGF‐A) and more strongly suppressed T cell proliferation to establish an immunosuppressive microenvironment in head and neck squamous cell carcinoma.^19^ Other studies showed that CAFs secreted macrophage colony‐stimulating factor to induce an M2 macrophage phenotype, which further promoted pancreatic tumour progression.^20^ Because of complex compositions of the TME, the relationship between CAFs and immune cells needs further study and analysis. The present review discusses interactions between CAFs and different immune cell types, their impact on tumour progression and potential therapeutic targets.

## 2 SOURCES AND MARKERS OF CANCER‐ASSOCIATED FIBROBLASTS

**Figure 1:**
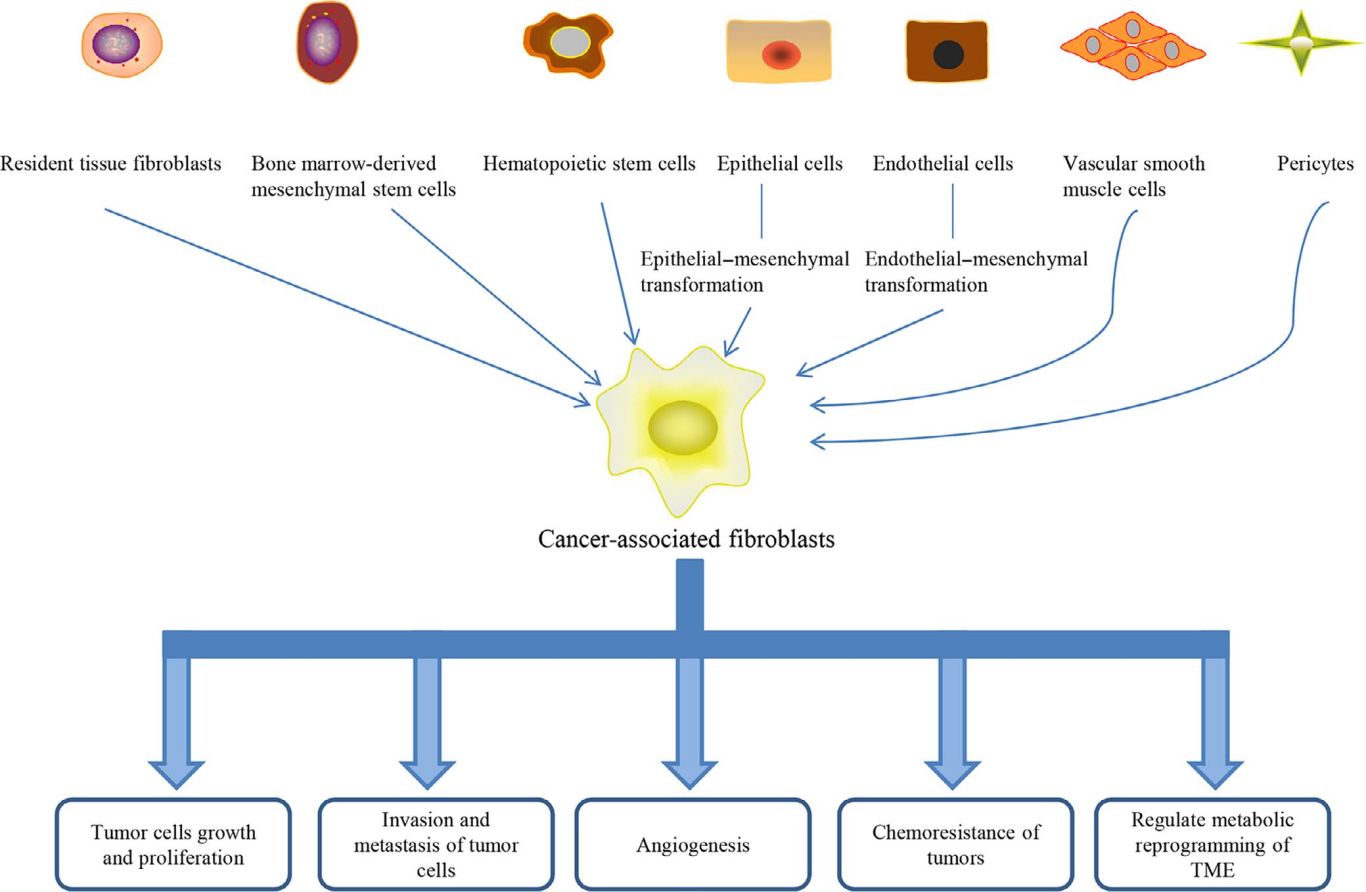
Origins of cancer‐associated fibroblasts and its roles in cancer progression. CAFs can originate from resident tissue fibroblasts, bone marrow‐derived mesenchymal stem cells, hematopoietic stem cells, epithelial cells, endothelial cells, vascular smooth muscle cells and pericytes. In the TME, CAFs can regulate cancer growth and proliferation, invasion and metastasis, angiogenesis, chemoresistance and metabolic reprogramming of the TME

Many theories have been proposed about the origins of CAFs, which is still controversial. For example, resident tissue fibroblasts, bone marrow‐derived mesenchymal stem cells (MSCs), hematopoietic stem cells, epithelial cells and endothelial cells are all considered possible precursors of CAFs, indicating that CAFs are heterogeneous^21^ (Figure 1). For example, Ronnov et al found that CAFs in breast cancer originated from residual fibroblasts, vascular smooth muscle cells and pericytes.^22^ A large proportion of CAFs appear to originate from the activation of resident tissue fibroblasts, which is constitutive and persistent.^23^ Several studies showed that the activation of fibroblasts is a reversible process.^23^, ^24^ Ren et al reported that the inhibitor of miR‐21, AC1MMYR2 (AMR), could reprogramme the breast cancer‐associated fibroblasts (BCAFs) into NFs.^24^ Furthermore, the transdifferentiation of pericytes, endothelial cells and epithelial cells can also produce a CAF‐like hybrid cell population that undergoes an endothelial–mesenchymal transformation 25 and epithelial–mesenchymal transformation.^26^ Moreover, studies have reported that MSCs could recruit and proliferate into myofibroblasts in the TME.^27^ Mesenchymal stem cells that derived from bone marrow acquired activated CAF phenotypes only in a conditioned medium of tumour cells.^28^ Because of the inherent heterogeneity of CAFs in cancer, inflammatory fibroblasts and myofibroblasts are two subpopulations of CAFs. Based on the distinct expression of α‐smooth muscle actin (α‐SMA), Öhlund et al found that fibroblast activating protein‐positive (FAP^+^) α‐SMA^high^ fibroblasts are myofibroblastic CAFs (myCAFs), and α‐SMA^low^IL‐6^high^ CAFs are inflammatory CAFs (iCAFs) in pancreatic tumours.^29^

**Figure 2:**
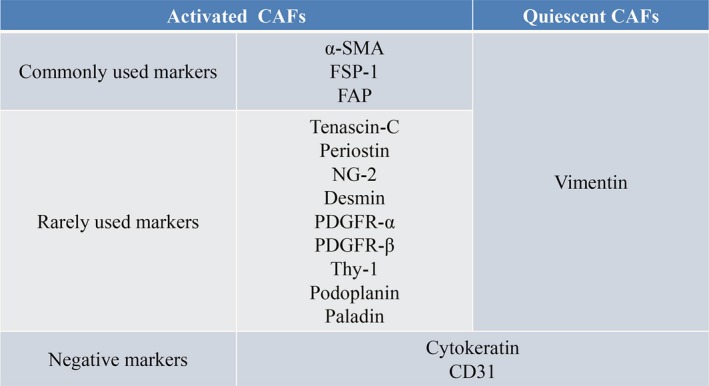
Markers of CAFs

Notably, that there is no precise molecular definition of CAFs, and CAFs tend to be a cellular state rather than a cell type.^30^ Importantly, CAFs need to be distinguished from normal fibroblasts.^31^ These activated fibroblasts can be identified according to molecular markers. Some commonly used markers, such as α‐SMA, fibroblast‐specific protein‐1 (FSP‐1 or S1001A4) and FAP 1, 3 (Figure 2). Studies have shown that quiescent fibroblasts express vimentin as a molecular marker. However, the most widely used marker in CAFs is α‐SMA, which may be because more myofibroblasts are in the tumour matrix.^32^ In fact, myofibroblasts are thought to be the same as CAFs, but not all CAFs express SMA. Thus, myofibroblasts are considered one subtype of CAF. Another common marker of CAFs is FAP, which is also a marker of myofibroblasts.^33^ However, the tissue distribution and function of FAP‐α is not limited to stromal fibroblasts, and expression can also be detected in epithelial malignant cells.^34^ Other studies have shown that tenascin‐C,^35^ periostin,^36^ glial cell antigen‐2 (NG‐2),^21^ desmin, platelet‐derived growth factor‐α and β (PDGFR‐α and ‐β), thy‐1 (CD90) and podoplanin can also be considered markers of CAFs.^37^ However, these markers are not necessarily specific to CAFs; they can also be expressed in other cells. For example, α‐SMA is also expressed in vascular myocytes. NG2 and PDGFR‐β are also expressed in normal pericytes, and podoplanin is expressed in lymphatic endothelial cells.^38^ Furthermore, Cannon et al found that paladin, a highly expressed actin binding protein in cancer, was expressed earlier than α‐SMA when activated in CAFs.^39^ Cytokeratin and CD31 are regarded as negative markers because CAFs had no epithelial or endothelial characteristics.^37^, ^40^ Because of the lack of specific CAF markers, combinations of markers may help identify CAFs. Combinations of morphological and markers are the most reliable way to identify CAFs.

## 3 CROSSTALK BETWEEN CANCER‐ASSOCIATED FIBROBLASTS AND IMMUNE CELLS

**Figure 3:**
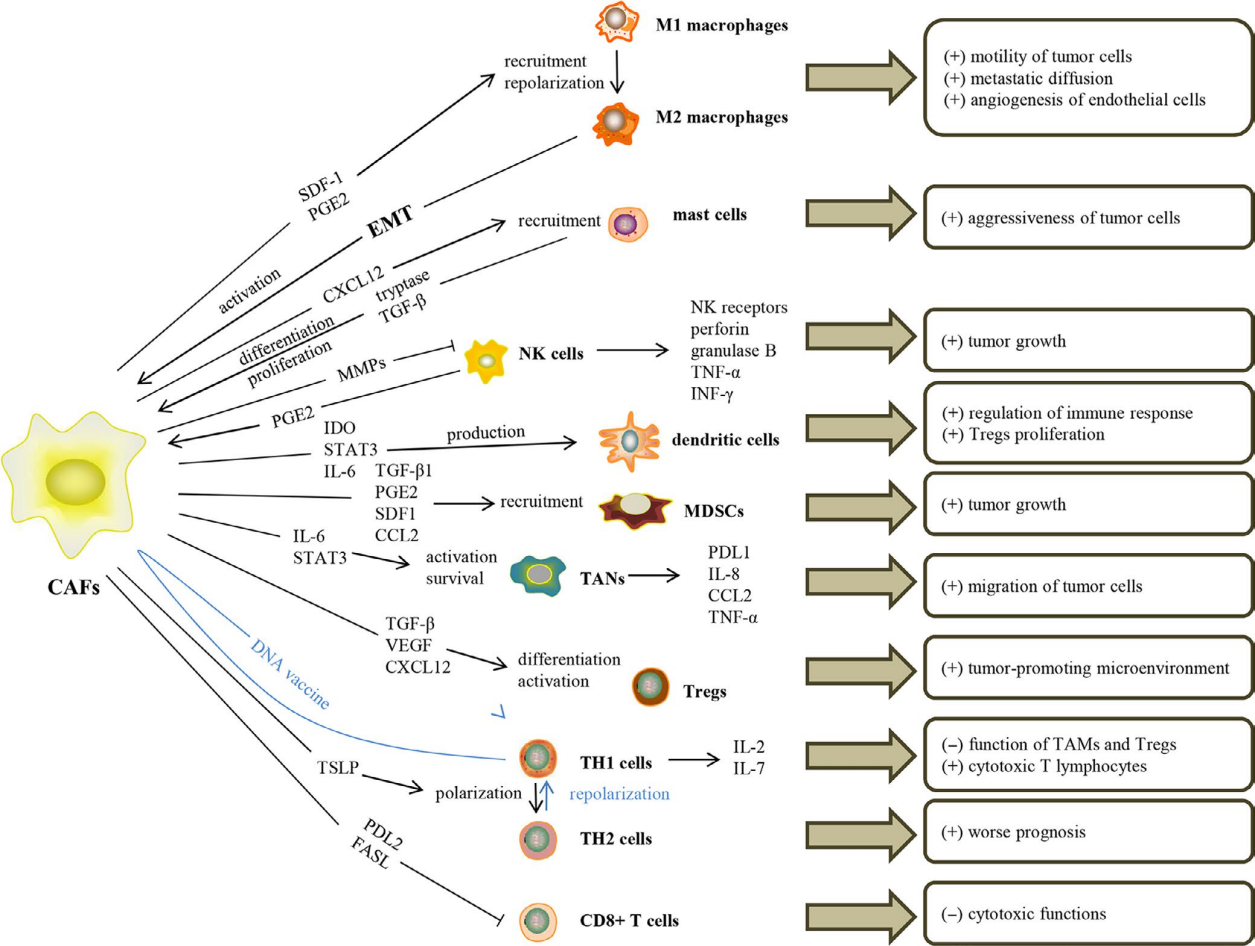
Crosstalk between cancer‐associated fibroblasts and immune cells, including macrophages, mast cells, NK cells, dendritic cells, MDSCs, TANs and T lymphocytes involved in the TME, and their functions in cancer progression

Inflammation is considered a marker of cancer and closely related to the reactivity of matrix fibroblasts. The relationship between inflammation and immune cells is inseparable. CAFs interact with immune cells and cancer cells via secreting cytokines, chemokines and other factors, such as collagens, MMPs, laminin, CXCL2, VEGF and TGF‐β.^41^ A large number of immune cells in cancer tissue have typically been associated with a better prognosis. However, accumulating evidence indicates that immune cells in cancer tissue do not play an anti‐tumour role but rather contribute to the occurrence and development of cancer.^42^ However, CAFs also play an important role in the TME Several studies have shown that immune cells interact with CAFs to regulate the TME 41, 43, 44 (Figure 3).

### 3.1 Interaction between cancer‐associated fibroblasts and macrophages

Numerous studies have shown that CAFs and macrophages interact to promote the progression of cancer. Some studies reported that accumulation of macrophages in TME correlated with poor prognosis of patients.^45^, ^46^ Mazur et al reported that type I collagen cleaved by FAP from activated fibroblasts, a post‐prolyl peptidase, could act as the substrate of macrophages which recognized by macrophages class A scavenger receptors (SR‐A); therefore, it increased the macrophages adhesion in cancer.^47^ Tumour‐associated macrophages (TAMs) consist of two groups with different phenotypes. M1 macrophages play an anti‐tumour role by activating the immune system and producing reactive oxygen species, nitric oxide and TNF. M2 macrophages perform immunosuppressive functions, promoting tumour progression^48^ and angiogenesis and degrading extracellular matrix.^49^ Accumulating evidence indicates that CAFs drive epithelial–mesenchymal transformation, maintain the growth of cancer cells and interact with M2 macrophages to promote the occurrence and progression of malignant tumours.^50^ Interestingly, TAMs and CAFs are often detected in the same area in tumour tissues. However, the proportions of these two cells are different in distinct tumours. In gastrointestinal cancer, lung cancer, pancreatic cancer and prostatic cancer tissues, CAFs expressed higher density than TAMs.^51^ However, in brain cancer, lymphoma, kidney cancer and hepatocellular carcinoma tissues showed higher density of TAMs.^51^ Gok et al reported that compared to NFs, CAFs could not only recruit monocytes via monocyte chemotactic protein‐1 (MCP‐1) and stromal cell‐derived factor‐1 (SDF‐1), but also differentiated monocytes into M2 macrophages with higher expression of IL‐10, therefore exerting immunosuppressive roles in breast cancer.^52^ In prostate cancer, CAFs are beneficial factors for monocyte recruitment to cancer cells, mainly by transferring stromal derived growth factor‐1 and promoting the transformation of macrophages into the M2 phenotype.^53^ This complex interaction between tumour cells, CAFs and M2 macrophages enhances the motility of tumour cells, thereby promoting escape from primary tumours, metastatic diffusion and the angiogenesis of endothelial cells. High levels of ERα expression in CAFs inhibited the invasion and migration of prostate cancer by affecting TAM infiltration in vitro and in vivo.^54^ ERα decreased the expression of chemokine (C‐C motif) ligand 5 (CCL5) and IL‐6 in CAFs and macrophages that were co‐cultured with CAFs in conditioned medium. These data suggest that ERα^+^ CAFs can be used as prognostic markers to predict the progression of prostate cancer. Kock et al found that the expression of prostaglandin E2 (PGE2), which was mainly secreted by CAFs, was blocked by the small‐molecular inhibitor compound III (CIII), which regulated the activity of microsomal prostaglandin E synthase‐1 (mPGES‐1). CIII inhibited tumour progression by shifting the M1/M2 ratio in neuroblastoma.^55^ Immunohistochemical staining and flow cytometry showed that CIII decreased the expression of CD206 and increased the ratio of M1/M2.^55^

Cancer‐associated fibroblasts regulate macrophages by secreting various cytokines. Macrophages can also regulate the status of CAFs. M2 macrophages affect the epithelial–mesenchymal transformation of fibroblasts. Fibroblasts that were activated by macrophages dynamically stimulated prostatic cancer, which were mediated by IL‐6 and SDF‐1.^53^ Zhang et al found that in a co‐culture with macrophages, MSCs that were derived from the human umbilical cord differentiated into CAFs, and promoted gastric epithelium cell malignancy via epithelial–mesenchymal transition (EMT).^56^ However, research that has focused on the influence of macrophages on CAFs has been very limited, and further studies are needed.

### 3.2 Interaction between cancer‐associated fibroblasts and mast cells

Studies of mast cells initially focused on asthma and allergic diseases. However, in recent decades, more studies have found that mast cells also participate in innate and adaptive immune‐related diseases, including cancer.^57^, ^58^, ^59^ As a hormone‐dependent tumour, CAFs in prostate tumours have a higher oestrogen receptor/androgen receptor (ER/AR) ratio compared with NFs. Ellem et al found that oestrogen‐induced CAF expression recruited mast cells to the TME by secreting CXCL12 in prostate cancer.^60^ Therefore, estrogens play an important role in mediating the interaction between CAFs and mast cells in prostate cancer.

Pereira et al found that mast cells secreted tryptase to promote the CAF‐induced transformation of prostate epithelia cell morphology in a micro‐tissue model.^61^ In ameloblastomas, the expression of myofibroblasts and mast cells was positively correlated, and this positive correlation was also correlated with the aggressiveness of cancer cells.^62^ This study showed that mast cells induced the differentiation fibroblasts into myofibroblasts and promoted myofibroblast proliferation.^62^ Yang et al found that mast cells and fibroblasts interact with each other to regulate the tumour phenotype in neurofibromatosis type 1 (NF1).^63^ NF+/− mast cells secreted TGF‐β, which promoted the proliferation of fibroblasts.^63^

### 3.3 Interaction between cancer‐associated fibroblasts and natural killer cells

Natural killer cells play an important role in tumour immunity. Li et al reported that CAFs significantly inhibited the function of NK cells in co‐culture experiments, but fibroblasts from normal skin had minimal effects on the phenotype and function of NK cells.^64^ In the co‐culture experiments, CAFs inhibited the expression of NK receptors, perforin and granulase B and inhibited secretion of the cytokines TNF‐α and interferon‐γ (IFN‐γ), suggesting that the inhibitory effect of CAFs on NK cells can be affected in different ways to promote tumour growth.^64^ Natural killer group 2 member D (NKG2D) is one of the activating receptors of NK cells, which is essential to the activation of NK cells. MICA/B work as the two ligands of NKG2D, express at the surface of tumour cells. Ziani et al showed that the increasing secretion of matrix metalloproteinases (MMPs) by CAFs in the melanoma tumour microenvironment could reduce the expression of MICA/B, therefore further decreasing the cytotoxic activity of NK cells to melanoma cancer cells which depended on NKG2D.^65^ Compared to the normal endometrial fibroblasts (NEFs), the expression of poliovirus receptor (PVR) was downregulated in CAFs.^66^ PVR is expressed on the cell surface of NEFs and CAFs, which is a ligand of the NK activating receptor DNAX accessory molecule‐1 (DNAM‐1). Therefore, CAFs could inhibit the killing activity of NK cells through downregulation PVR on the cell surface, which further promoted cancer development.^66^ Additionally, Zhang et al reported that CAFs secreted IL‐8 to attract monocytes and promoted M2 macrophages polarization.^67^ Furthermore, the synergetic effects of CAFs and TAMs, which induced by CAFs increasing the suppression of NK cells functions in colorectal cancer.^67^ In addition, CAFs significantly suppressed the cytotoxicity function of NK cells via releasing PGE2 in melanoma.^68^

In the presence of NK cells, the CAF‐induced expression of PGE2 was higher than fibroblasts,^64^ suggesting that NK cells can also influence the cytokine expression of CAFs. However, studies of the influence of NK cells on CAFs are limited, and further studies are needed.

### 3.4 Interaction between cancer‐associated fibroblasts and dendritic cells

Dendritic cells (DCs) are effective antigen‐expressing cells that can stimulate the primary immune response by expressing class I and class II MHC complexes, co‐stimulatory molecules and adhesion molecules, which can stimulate the immature T cell population.^69^ Cancer‐associated fibroblasts play a key role in immune regulation, but their role in regulating dendritic cells remains unclear. Cheng et al reported that CAFs that were derived from hepatocellular carcinoma promoted the production of regulatory DCs, which was associated with low co‐stimulatory molecule expression, high suppressive cytokines production and the enhancement of regulation of the immune response, including the proliferation of Treg cells through the upregulation of indoleamine‐2,3‐dioxygenase (IDO).^70^ This research also showed that IL‐6 that was derived from CAFs was necessary for the generation of IDO. Additionally, IDO inhibitors, anti‐signal transducer and activator of transcription 3 (STAT3) and anti‐IL‐6 antibodies reversed the regulatory effect of CAFs on DCs.^70^ Several studies have shown that IDO is highly expressed in regulatory DCs in tumour areas, which can inhibit the anti‐tumour immune response.^70^, ^71^, ^72^ IDO participates in the immune tolerance and suppression of tumour cells by inducing T cell anaemia and Treg cell proliferation. Therefore, the interaction between CAFs and DCs is complex and highly correlated with the T cell immune response.

### 3.5 Interaction between cancer‐associated fibroblasts and myeloid‐derived suppressor cells

Myeloid‐derived suppressor cells (MDSCs) are a group of heterogeneous cells that are derived from bone marrow. They are precursors of DCs, macrophages and granulocytes and have a remarkable ability to suppress immune cell responses. The CAFs inhibitor tranilast decreased the expression of TGF‐β1, PGE2 and SDF1, thus resulting CAFs suppressed regulatory T cells and MDSCs.^73^ CAFs could attract monocytes and further differentiate them into MDSCs via activating STAT3 mediated by IL‐6, which creating an immunosuppression microenvironment by inhibiting T cell proliferation in hepatocellular carcinoma.^74^ Yang et al reported that FAP^+^ CAFs secreted CCL2 to promote tumour growth via the recruitment of MDSCs in a murine liver tumour model.^75^ Multiple studies have shown an anti‐tumour effect of inhibiting the colony‐stimulating factor 1 receptor (CSF1R). Vinit et al found that CSF1R inhibition decreased TAMs, which was correlated with the recruitment and accumulation of polymorphonuclear myeloid‐derived suppressor cells (PMN‐MDSCs) in tumours, and an increase in the secretion of CXCL‐1 by CAFs.^76^ This demonstrated a potential correlation between CAFs and MDSCs. Further studies showed that the inhibition of CXCR2 (a major CXCL‐1 receptor that is expressed by granulocytes), combined with the inhibition of CSF1R reduced both TAMs and PMN‐MDSCs in tumours, which significantly inhibited tumour growth.^76^ Moreover, such inhibition further enhanced the tumour inhibition ability of PD‐1 antibody.

Circulating fibrocytes were reported to be a subset of MDSCs that are involved in regulating tumour immune escape.^77^ However, CAFs appear to be similar to circulating fibrocytes, with few differences in molecular expression. Fibrocytes were shown to express both MHC‐II and CD80/86, whereas CAF only expressed MHC‐II. CD11b was reported to be expressed by murine and human MDSCs, and CAFs were also mildly positive for CD11b/c.^77^ This suggests that CAFs might have a similar function to MDSCs.

### 3.6 Interaction between cancer‐associated fibroblasts and tumour‐associated neutrophils

Studies that have focused on tumour‐associated neutrophils (TANs) are very limited. In hepatocellular carcinoma (HCC), CAFs promoted the activation and survival of neutrophils through the IL6/STAT3/programmed death ligand 1(PDL1) signalling pathway.^78^ In co‐culture with CAFs, TANs expressed more PDL1, IL‐8, CCL2 and TNF‐α.^78^ Neutrophils that were activated by CAFs regulated the STAT3‐PDL1 pathway to impair the immunity function of T cells in HCC.^78^ Zhu et al reported that neutrophils that were activated by MSCs promoted the normal transformation of MSCs into CAFs in gastric cancer with high expression of FAP, which further promoted the migration of gastric cancer cells.^79^

### 3.7 Interaction between cancer‐associated fibroblasts and T lymphocytes

Several studies have shown that Tregs are more abundant in stroma than in the cancer nest. Patients with higher Tregs expression had a worse prognosis than patients with lower Tregs expression.^80^ Berna et al reported that the number of CD4^+^Foxp3^+^ T‐regulatory cells (Tregs) was higher in myofibroblast‐depleted mice with pancreatic cancer, which exhibited the suppression of angiogenesis and promotion of EMT.^81^ However, the depletion of myofibroblasts promoted tumour invasion, thus leading to a decrease in overall survival time in mice. Worse survival and poorly differentiated tumours were correlated with lower SMA^+^ expression in patients with pancreatic ductal adenocarcinoma (PDAC).^81^ In contrast to the traditional view that CAFs promote tumour progression, this study found that the presence of myofibroblasts was correlated with immunotherapy and a better prognosis of PDAC patients. Additionally, CAFs in adenocarcinomas with higher Tregs expression had higher TGF‐β and VEGF expression.^80^ Histochemistry confirmed that most TILs, including Tregs cells, were located in the cancer matrix and adjacent to CAFs. These findings suggested that these two cells play a role in the TME.^80^ Immunoregulatory cytokines expression in CAFs may induce Tregs in the matrix, create a tumour‐promoting microenvironment in lung adenocarcinoma, and result in a worse prognosis.^80^

Interestingly, Costa et al found that because of the heterogeneity of CAFs in breast cancer, CAF‐S1 and CAF‐S4 represented two subsets of myofibroblasts.^82^ This study found that CAF‐S1 was correlated with CD25^+^FOXP3^+^ lymphocytes and promoted the attraction of CD4^+^CD25^+^ T lymphocytes via the secretion of CXCL12, thus promoting formation of an immunosuppressive environment.^82^ Importantly, CAF‐S1 induced Tregs differentiation and activity, but CAF‐S4 did not exhibit these properties.^82^ Therefore, CAFs from different origins exert different biological functions, especially with regard to effects on Tregs.

Helper T cells (Th cells) are mainly divided into Th1 cells and Th2 cells. Th1 cells participate in cellular immunity and a delay in hypersensitivity inflammation. Th2 cells can assist B cells to differentiate into antibody‐secreting cells and participate in the humoral immune response. Th cells commonly express CD4. Therefore, CD4^+^ T cells mainly imply Th cells. Several studies have shown that CAFs play an important role in regulating the balance between Th1 and Th2 cell expression. TNF‐α and IL‐1β that were secreted by tumours activated CAFs, which further secreted thymic stromal lymphopoietin (TSLP) in pancreatic cancer.^83^ This could cause Th2 cell polarization and result in a worse prognosis. Liao et al found that targeting FAP^+^ CAFs with a DNA vaccine promoted the transition of polarization from Th2 cells to Th1 cells, which increased the expression of IL‐2 and IL‐7, suppressed the function of TAMs and Tregs, and activated cytotoxic T lymphocytes (CTLs) in primary breast cancer.^84^

CD8^+^ T cells are usually referred to as T cytotoxic (Tc) cells, which are further differentiated and proliferate into effector cells after activation, called CTLs. Cytotoxic T lymphocytes are a special type of T cell that can kill some antigens, such as viruses and cancer cells, and form an important line of defence against viruses and cancer with NK cells. Lakins et al reported that CAFs suppressed CD8^+^ T cells via PDL2 and FASL. Therefore, the inhibition of PDL2 and FASL in CAFs could reactivate cytotoxic T cells.^85^ Kato et al reported that co‐cultured colon cancer cells with CAFs increased the expression of FoxP3^+^ TILs (regulatory T cells), while decreased the expression of CD8^+^ TILs (cytotoxic T cells).^86^ This was due to CAFs secreted high levels of IL‐6. The blockade of IL‐6 could not only inhibit tumour growth, but also promote the accumulation of CD8^+^ TILs in tumour.

## 4 REGULATION OF CANCER‐ASSOCIATED FIBROBLASTS IN CANCER

Cancer‐associated fibroblasts play an important role in cancer. The crosstalk between cancer cells and TME, especially stroma cells is widely discussed, which might be the major cause of tumour progression.^87^ Fu et al showed CAFs could secrete and produce the energy metabolites, such as pyruvate and ketone bodies, in order to supply cancer cells.^88^ Cancer‐associated fibroblasts stimulate the growth and proliferation of tumour cells. Matthew et al found that CAFs activated an immune checkpoint to suppress the function of T cells, which was mediated by the engagement of PDL2 and FASL Therefore, CAFs contribute to the suppression of anti‐tumour T cell response by regulating immune cells, such as promoting antigen‐specific T cells death.^85^ Cancer‐associated fibroblasts can also promote the invasion and metastasis of tumour cells. Cancer‐associated fibroblasts produce the tryptophan metabolite kynurenine and were shown to inhibit the differentiation of DCs and promote tumour cell growth and migration in lung cancer.^9^ Galectin‐1 upregulated tryptophan 2,3‐dioxygenase (TDO2) expression in CAFs. The inhibition of TDO2 decreased tumour metastasis by promoting the T cell response in vivo.^9^ Moreover, metalloproteinases that were secreted by CAFs promoted the release of Ras‐related C3 botulinum toxin substrate (Rac1b)/cyclooxygenase 2 (COX‐2)‐mediated reactive oxygen species in cancer cells, which is essential for EMT, cell stemness and metastasis.^89^ Additionally, CAFs promote angiogenesis through a complex interaction with cancer cells and macrophages. Comito et al reported that polarized M1 macrophages were transformed into M2 polarization de novo by CAFs and prostate carcinoma (PCa) cells, which further drove the vascularization of PCa cells.^53^ Moreover, CAFs regulate the chemoresistance of tumours. Wang et al reported that stromal fibroblasts promoted chemoresistance by decreasing the nuclear accumulation of platinum in ovarian cancer, which was mediated by glutathione and cysteine.^90^ CD8^+^ T cells reversed this chemoresistance through IFN‐γ the via JAK/STAT1 pathway.^90^ Finally, CAFs regulated metabolic reprogramming of the TME. By targeting nuclear transcription factors, p62 repressed tumour progression by regulating metabolic reprogramming.^91^ In fact, p62 deficiency in stroma promoted the upregulation of ATF4, thus further promoting tumour proliferation by generating asparagine, which served as a source of nitrogen for cancer.^91^ In addition, several researches have shown that exosomes from CAFs could promote the tumour progression.^92^, ^93^ Richards et al found that exosomes from CAFs significantly increased the chemoresistance to gemcitabine in pancreatic ductal adenocarcinomas (PDACs).^92^ Li et al reported that exosomes from CAFs increased the expression of TGF‐β1, which further enhanced the migration and invasion ability via SMAD signalling pathway in ovarian cancer.^93^

## 5 ANTI‐CANCER‐ASSOCIATED FIBROBLAST THERAPIES

Fibroblasts from different parts of the body and organs have different characteristics, including their susceptibility to acquire CAFs phenotypes and interactions with adjacent epithelial cells and immune cells.^94^ In contrast to cancer epithelial cells, genetic changes in CAFs (eg changes in copy number or mutations of oncogenes or tumour suppressor genes) are extremely rare.^30^, ^95^ Therefore, the promotion of tumours by CAFs does not appear to result from genetic alterations. Anti‐CAFs therapies, including many drugs that target FAP, have been shown to exert significant anti‐tumour effects in pre‐clinical models.^96^ High concentrations of curcumin were shown to have cytotoxic effects on CAFs. However, a low concentration of curcumin had few effects on the proliferation of CAFs but decreased the expression of α‐SMA and vimentin, suggesting that curcumin at low concentrations can reverse the activation of fibroblasts.^97^ Conditioned medium with CAFs increased the migration and invasion ability of pancreatic cancer cells, and conditioned medium with curcumin‐treated CAFs had minimal effects on the migration ability of pancreatic cells.^97^ Prostate cancer cells were treated with CAF‐conditioned medium and flavonoid silibinin‐conditioned medium. The CAF‐conditioned medium significantly reduced the expression of E‐cadherin in cancer cells and increased the invasiveness of cancer cells.^98^ Monocyte chemo‐attractant protein‐1 (MCP‐1) is a key component in promoting cancer cell invasion. Cancer‐associated fibroblasts that were treated with the flavonoid silibinin exhibit lower expression of the MCP‐1 transcription regulators nuclear factor κB (NF‐κB) and AP‐1, consequently decreasing the invasiveness of cancer cells.^13^, ^98^ Studies have shown that the overexpression of FAPα promotes the growth and metastasis of tumours. Treatment with anti‐FAPα antibodies or pulsed DCs inhibited tumour growth.^99^ To destroy specific CAFs, Xia et al constructed a DNA vaccine that expressed human FAPα. The vaccine successfully reduced the growth of 4T1 tumours by producing a specific CTL response to FAPα that killed CAFs.^100^ FAPα‐based vaccines may be used to induce FAPα‐specific CTLs to kill CAFs and destroy immunosuppressive components in the TME.^101^, ^102^ These vaccines can also reduce the risk of immune escape, which is an advantage that tumour‐associated antigen (TAA) does not have. Liao et al showed that FAP was specifically overexpressed in the fibroblasts in the tumour stroma.^84^ CD8^+^ T cells could exert cytotoxic effect to the tumour via targeting the FAP antigen in the CAFs.^103^ Therefore, these studies showed that the targeting CAFs via FAP antigen can inhibit tumour growth and progression, which possibly mediated by T cell immunotherapy.^33^ A DNA vaccine that targeted the tumour matrix antigen FAPα induced an anti‐tumour immune response that was mainly mediated by CD8^+^ T cells.^104^

**Figure 4:**
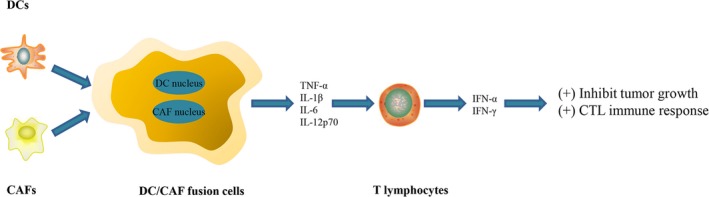
Fusion cells generated with dendritic cells and cancer‐associated fibroblasts, which further regulate T lymphocytes with higher expression of IFN‐α and IFN‐γ

Dendritic cells function as powerful antigen‐presenting cells (APCs) to elicit a strong immune response to tumour antigens. However, the invasion of DCs in the tumour host is lower, and the function of DCs is impaired. In vitro cultures of DCs that contained tumour antigen and DC vaccines may be effective strategies for achieving a strong immune response of the tumour host (Figure 4). However, DC vaccines against cancer cells have been shown to have only limited anti‐tumour activity in most clinical studies. Studies have shown that targeting CAFs can enhance the anti‐tumour effect. The fusion of DCs and CAFs can stimulate T cells to inhibit tumour growth. Co‐cultures of DCs from bone marrow from BALB/c mice and CAFs from stem cells from H22 mice presented increases in the secretion of TNF‐α, IL‐1β, IL‐6 and IL‐12p70, which effectively stimulated the production of T lymphocytes in vitro, and induced the production of IFN‐α and IFN‐γ by T lymphocytes.^105^ Therefore, T cells that are activated by DC/CAF fusion cells can induce a strong CTL immune response to CAFs in vitro. These results suggest that DC/CAF fusion cells can stimulate T cells and may serve as a new type of anti‐tumour immune vaccine.^105^ Gottschalk et al developed a new compound DC vaccine (DC‐shA20‐FAP‐TRP2) that targeted FAP and tumour antigen tyrosine‐related protein 2 (TRP‐2).^106^ The vaccine enhanced the tumour infiltration of CD8^+^ T cells and induced antigen diffusion, leading to effective anti‐tumour activity.^106^ Mice that were treated with anti‐CAF exhibited lower TGF‐β expression in the TME. In mice that were immunized with the DC vaccine and anti‐CAF, the expression of TGF‐β was significantly reduced, which effectively reduced Tregs.^107^ Additionally, reducing SDF‐1 expression by inhibiting CAFs may be related to a decrease in Tregs migration in the TME, in which blockade of the CXCR4‐CXCL12 axis prevented Tregs from migrating to the TME.^108^ When combined with anti‐CAF therapy, the efficacy of the DC vaccine increased, which contributed to the effective inhibition of tumour growth and reduced the level of immunosuppressive cytokines in tumour tissues. Furthermore, Yasuhiko et al reported that the inhibition of CAFs in SCID mice did not influence tumour growth but modulated the immune function in the TME.^107^

In addition to basic research, some clinical studies have targeted CAFs. The activation of hedgehog (Hh) signalling is important for CAF function. In a phase I clinical trial (NCT02027376), Aurélie et al used docetaxel with smoothened inhibitors (SMOi), which inhibited hedgehog signalling in CAFs for the treatment of TNBC patients and improved outcomes.^109^ Some patients even presented a complete clinical response (ie disappearance of the tumour lesions without new lesions).^109^ In a phase I clinical trial, the antibody targeting of FAP with repeated infusions of sibrotuzumab was shown to be useful for the treatment of advanced FAP^+^ cancer patients.^110^ It is important for clinicians to find the antigens which specifically expressed in tumour cells or tumour stroma with minimum expressed in normal tissues. Fibroblast activation protein (FAP) is a type II membrane‐bound glycoprotein, which expressed on the activated CAFs in tumour stroma instead of normal tissues.^111^ The blockade of FAP could inhibit the ability of CAFs in promoting cancer cells invasion and metastasis. Sibrotuzumab is the humanized antibody targeting FAP By using [^131^I]sibrotuzumab, researchers found that the antibody mainly uptakes in the tumour sites instead of normal tissue sites.^110^ Therefore, the advantage of targeting FAP is sibrotuzumab could successfully uptake in tumour sites because of FAP is not widely expressed in normal tissues antigen pool. Thus, sibrotuzumab had high tumour specificity.^110^ However, this study only focused on the ability of sibrotuzumab to target FAP and did not evaluate the efficacy of sibrotuzumab to treat patients. One patient presented stable disease status, but further clinical studies are needed. Few clinical trials have focused on CAFs and immune cells.

We still have a long way to go to translate in vitro studies to in vivo studies and clinical trials. Several studies have shown that targeting anti‐CAFs may be a promising therapeutic approach. Walter et al found that the sonic hedgehog (SHH) signalling pathway is overactivated in pancreatic ductal adenocarcinoma (PDAC), and this overactivation stimulates CAFs to regulate matrix protein production. PDAC cells produce SHH ligands that bind to receptor patched‐2 on CAFs.^112^ However, in a phase II clinical trial (NCT01064622), the combination of gemcitabine with vismodegib (ie an inhibitor of the hedgehog pathway) did not improve overall survival or progression‐free survival in pancreatic cancer patients.^113^ This treatment failure may be attributable to an inappropriate therapeutic combination. Several studies showed that the inhibition of Hh is caused by IPI‐296, which is also known as saridegib.^114^ As an anti‐FAP monoclonal antibody, unconjugated sibrotuzumab BIBH1 may be a potentially useful immunological‐related antibody to target FAP for the treatment of colon cancer,^115^ but no complete or partial response was observed in this study. Only two of 17 patients presented stable status in advanced metastasis colorectal cancer. This treatment failure may be attributable to the possibility that the unconjugated antibodies could not achieve a sufficient effect against solid tumours.^116^ Narra et al found that as the Val‐boroPro (Talabostat; an inhibitor of FAP enzymatic activity) inhibited tumour progression in metastatic colorectal cancer in a phase II trial.^96^ Only six of 28 patients presented stable disease status, thus indicating minimal clinical efficacy. This may be attributable to the incomplete inhibition of enzymatic activity. Other studies have shown that stromal pathways exert effects mainly in early stages of the disease, which may be another reason for treatment failure.^117^

## 6 CONCLUSIONS

Researches on CAFs in cancer and its targeted therapy may improve the prognosis of cancer patients. The interaction between CAFs and macrophages, mast cells, NK cells, DCs, MDSCs, TANs and T lymphocytes has a considerable effect on the regulation of tumour progression. As mentioned above, CAFs can interact with immune cells and cancer cells via various signalling pathway, such as autocrine, paracrine mechanisms and direct action, particularly the interaction between CAFs and cancer cells could through the secretion of exosomes, to form a complex molecular network and play its biological functions. Despite the increasing research on CAFs, it is noteworthy that large‐scale randomized clinical trials are still a major gap in targeted CAFs treatment. Many pre‐clinical studies have not shown significant anti‐tumour effects, nor can they significantly prolong the survival of patients. In the future, a large number of original studies targeting CAFs are needed to further elucidate their clinical value and impact on cancer progression.

## 7 ETHICAL APPROVAL AND CONSENT TO PARTICIPATE

This review does not contain any studies with human participants or animals performed by any of the authors.

## CONFLICTS OF INTERESTS

The authors have declared that no competing interest exists.

## AUTHORS’ CONTRIBUTIONS

Qing Yang designed the review. Yuanyuan An wrote the manuscript. All the figures were prepared by Yuanyuan An and revised by Qing Yang. Fengtian Liu and Ying Chen contributed to revise the manuscript.

## Data Availability

The data used to support the findings of this study are included within the article.
